# Gamma-oryzanol Prevents LPS-induced Brain Inflammation and Cognitive Impairment in Adult Mice

**DOI:** 10.3390/nu11040728

**Published:** 2019-03-29

**Authors:** Andrea Mastinu, Sara Anna Bonini, Wiramon Rungratanawanich, Francesca Aria, Mariagrazia Marziano, Giuseppina Maccarinelli, Giulia Abate, Marika Premoli, Maurizio Memo, Daniela Uberti

**Affiliations:** Department of Molecular and Translational Medicine, University of Brescia, 25123 Brescia, Italy; andrea.mastinu@unibs.it (A.M.); sara.bonini@unibs.it (S.A.B.); w.rungratanawani@unibs.it (W.R.); f.aria@unibs.it (F.A.); m.marziano@unibs.it (M.M.); giuseppina.maccarinelli@unibs.it (G.M.); m.premoli002@unibs.it (M.P.); maurizio.memo@unibs.it (M.M.); daniela.uberti@unibs.it (D.U.)

**Keywords:** γ-oryzanol, cognitive performance, neuroinflammation, second-generation antioxidant enzymes

## Abstract

Background: Rice (*Oryza sativa* L.) is the main food source for more than half of humankind. Rice is rich in phytochemicals and antioxidants with several biological activities; among these compounds, the presence of γ-oryzanol is noteworthy. The present study aims to explore the effects of γ-oryzanol on cognitive performance in a mouse model of neuroinflammation and cognitive alterations. Methods: Mice received 100 mg/kg γ-oryzanol (ORY) or vehicle once daily for 21 consecutive days and were then exposed to an inflammatory stimulus elicited by lipopolysaccharide (LPS). A novel object recognition test and mRNA expression of antioxidant and neuroinflammatory markers in the hippocampus were evaluated. Results: ORY treatment was able to improve cognitive performance during the neuroinflammatory response. Furthermore, phase II antioxidant enzymes such as heme oxygenase-1 (HO-1) and NADPH-dehydrogenase-quinone-1 (NQO1) were upregulated in the hippocampi of ORY and ORY+LPS mice. Lastly, γ-oryzanol showed a strong anti-inflammatory action by downregulating inflammatory genes after LPS treatment. Conclusion: These results suggest that chronic consumption of γ-oryzanol can revert the LPS-induced cognitive and memory impairments by promoting hippocampal antioxidant and anti-inflammatory molecular responses.

## 1. Introduction

**Figure 1:**
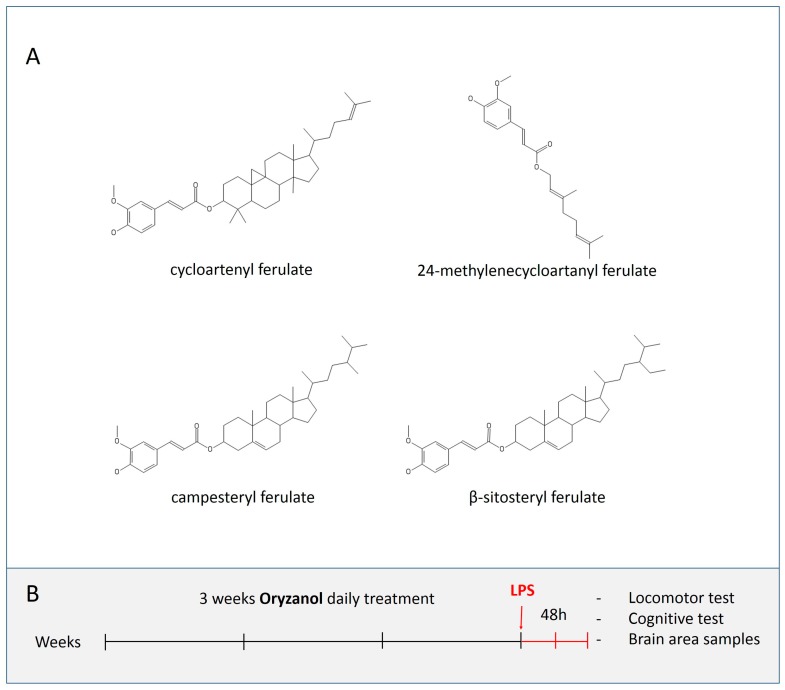
Chemical structures of different organic molecules contained in γ-oryzanol (**A**). Graphic scheme of γ-oryzanol and lipopolysaccharide (LPS) treatment protocol (**B**).

The genus *Oryza* includes about 23 species, among which rice (*Oryza sativa* L.) is one of the major sources of nutrition for about two-thirds of humankind [1,2]. Although two subspecies (*indica* and *japonica*), mainly grown in Japan, Korea, and northern China, have been identified, a phylogenetic analysis unravelled five distinct subpopulations of *Oryza sativa* L., two in *indica* (*indica* and *aus*) and three in *japonica* (*temperate japonica*, *tropical japonica*, and *aromatic*) [3]. In addition, *Oryza glaberrima* Steud is another endemic species cultivated in West Africa [4]. *O. sativa*, besides being one of the main source of complex carbohydrates in different diets, contains many bioactive compounds including vitamins, polyphenols, and minerals [5]. In particular, rice bran is rich in γ-oryzanol (ORY), an antioxidant polyphenol whose quantity and quality changes according to the rice variety and cultivar [6,7,8,9,10]. ORY extracted from rice bran is actually a mixture of several organic molecules (Figure 1A) consisting mainly of cycloartenyl ferulate, 24-methylenecycloartanyl ferulate, campesteryl ferulate, and β-sitosteryl ferulate, which are all ferulate esters of phytosterols [11]. The biological benefits of ORY have been intensively studied. In particular, its antioxidant activities and its beneficial properties, including anti-inflammatory and hypocholesterolemic effects, have been well documented [12,13,14]. Moreover, ORY is recognized as a good dietary supplement and a pharmaceutical candidate for the maintenance and enhancement of human health during ageing [15]. Thanks to its lipophilic characteristics, ORY passes through the blood-brain barrier and exerts effects even in the central nervous system (CNS) [16]. For example, in mouse models of anxiety and stress, Kozuka and colleagues demonstrated that ORY exerted its therapeutic effects by modulating dopamine receptor 2 [17]. Nevertheless, only few works have described the effects of ORY on cognitive functions, and its mechanism of action remains still unclear.

The main objective of this study was to investigate the effects of ORY in an in vivo model of neuroinflammation elicited by lipopolysaccharides (LPS). In particular, we explored the cognitive performance and the gene expression of inflammatory mediators as well as the phase II antioxidant enzymes in mice that were chronically treated with ORY and then challenged with an acute inflammatory insult.

## 2. Materials and Methods

### 2.1. Animals

Thirty-seven male B6/129PF2 mice (aged 12–14 months, average weight: 43 g) were purchased from The Jackson Laboratories (Bar Harbor, ME, USA). The mice were housed three-to-four per cage in a 12 h light/dark cycle (light phase from 8:00 a.m. to 8:00 p.m.) and were maintained at 50% relative humidity and a 12 h light/dark cycle at 20–22 °C. Mice were fed normal weighted chow (70% carbohydrate, 20% protein, 10% fat, total 3.95 kcal/g; standard diet 4RF21 (Mucedola, Milan, Italy) and water *ad libitum*. All experiments were conducted in conformity with the European Communities Council Directive of 1986 (86/609/EEC), approved by the Italian Ministry of Health and the Animal Care and Use Committee of the University of Brescia.

### 2.2. Chemicals and Treatment

Mice were weighed, divided into 2 groups and exposed to 3 weeks of chronic treatment with ORY (Sigma-Aldrich, Merck KGaA, Darmstadt, Germany) (20 mice) or a vehicle (VH, 17 mice). The vehicle consisted of a physiological solution (0.9% NaCl) (Sigma-Aldrich, Merck KGaA, Darmstadt, Germany). ORY and VH were administered by oral gavage once per day (Figure 1B). The dosage of ORY (100 mg/kg) was chosen according to previously reported data [18]. Individual body weights and cage food consumption were recorded weekly. At the end of the 21 days of treatment, 10 mice treated with ORY and 9 mice treated with VH received intraperitoneally (i.p.) 10 µg/mouse of lipopolysaccharide (LPS, Sigma-Aldrich, Merck KGaA, Darmstadt, Germany). After 48 h, mice were placed in the behavioural room for 30 min to acclimatize before each test was performed. In the behaviour room, the behavioural test area was separated from the operator by a dark sliding door. Rotarod and novel object recognition tests were executed as reported below.

### 2.3. Rotarod Test

To measure motor performance, mice were placed one by one on the rotarod treadmill (Ugo Basile, Varese, Italy), and a trial of 30 s at a constant speed of 2 rpm was executed. Immediately afterwards, the test was performed at an initial intensity of 2 rpm, and a final intensity of 20 rpm was reached 300 s later. Finally, the time that each rodent managed to stay on top of the rotarod treadmill was recorded.

### 2.4. Novel Object Recognition

To establish the cognitive performance of the mice in terms of long-term memory, the novel object recognition (NOR) test was used. This protocol was executed over three consecutive days, as previously reported [19,20], in a 40 × 40 cm Plexiglas square arena, and the average latency between the test phases was 24 h. On Day 1, the mice were allowed to acclimatize to an empty arena for 5 min. On Day 2, the mice were exposed to two identical objects for a 5-min period. On Day 3, one of the objects was replaced with a new object, and mice were let free to explore for 5 min. Each trial was recorded, and videos were analyzed with EthoVision XT software (Noldus IT, Wageningen, The Netherlands). The lengths of time spent with the familiar object and the novel object were calculated.

### 2.5. Gene Expression

After the behavioural tests, mice were sacrificed, and their hippocampi were isolated for mRNA analysis. The total RNA was extracted using the TRIzol^®^ Reagent (Sigma-Aldrich, Merck KGaA, Darmstadt, Germany), and two micrograms of total mRNA were reverse-transcribed using M-MLV reverse transcriptase (Promega, Madison, WI, USA) following the manufacturer’s instructions. Table 1 shows the murine-specific primers used for q-PCR. Amplification and detection were performed with the ViiA7 Real Time PCR Detection System (Applied Biosystems, Foster City, CA, USA). The reaction mix contained 6 μL of SYBR Green Master Mix (Bio Rad Laboratories, Richmond, CA, USA), 6 pmol of each forward and reverse primer, and 2 μL of diluted cDNA. The samples were run in duplicate, and the PCR program was initiated by 10 min at 95 °C before 40 cycles, each of 1 s at 95 °C and 30 s at 64 °C. The gene expression levels were normalized to β-Actin expression, and the data are presented as the fold change in target gene expression. Relative quantification was performed using the comparative Ct method.

### 2.6. Statistical Analysis

Two-way Repeated Measures ANOVA tests with the Bonferroni post-test were used to determine the significance of the pharmacological effect on body weight, food intake during the chronic treatment, and novel object recognition. One-way ANOVA tests with the Newman-Keuls post-test were instead adopted to determine the statistical difference for the rotarod test and the molecular analysis after the ORY and LPS treatments. Data are presented as the means ± S.E.M. (standard error mean). All statistical analyses were performed using GraphPad Prism version 6 (GraphPad Software, San Diego CA, USA), and the statistical significance level was set at *p* < 0.05.

## 3. Results

### 3.1. γ-oryzanol has No Effect on Mouse Body Weight or Food Intake 

The effects of chronic treatment with ORY were initially assessed in regard to the mice’s vital parameters, including body weight and food intake. At the beginning of the experiment, the group of mice (*n* = 20) tagged ORY and those labelled vehicle (VH) (*n* = 17) showed comparable body weights (43.28 ± 1.42 g) (Table 2). Three weeks of ORY treatment did not affect body weight or food intake (Table 2).

### 3.2. γ-oryzanol Prevents LPS-induced Cognitive Impairment

In order to evaluate the effects of ORY on LPS-induced cognitive impairment, ORY or VH pre-treated mice were injected intraperitoneally with 10 µg/mouse LPS, and 48 h later, they were subjected to behavioral tests (Figure 1B).

**Figure 2:**
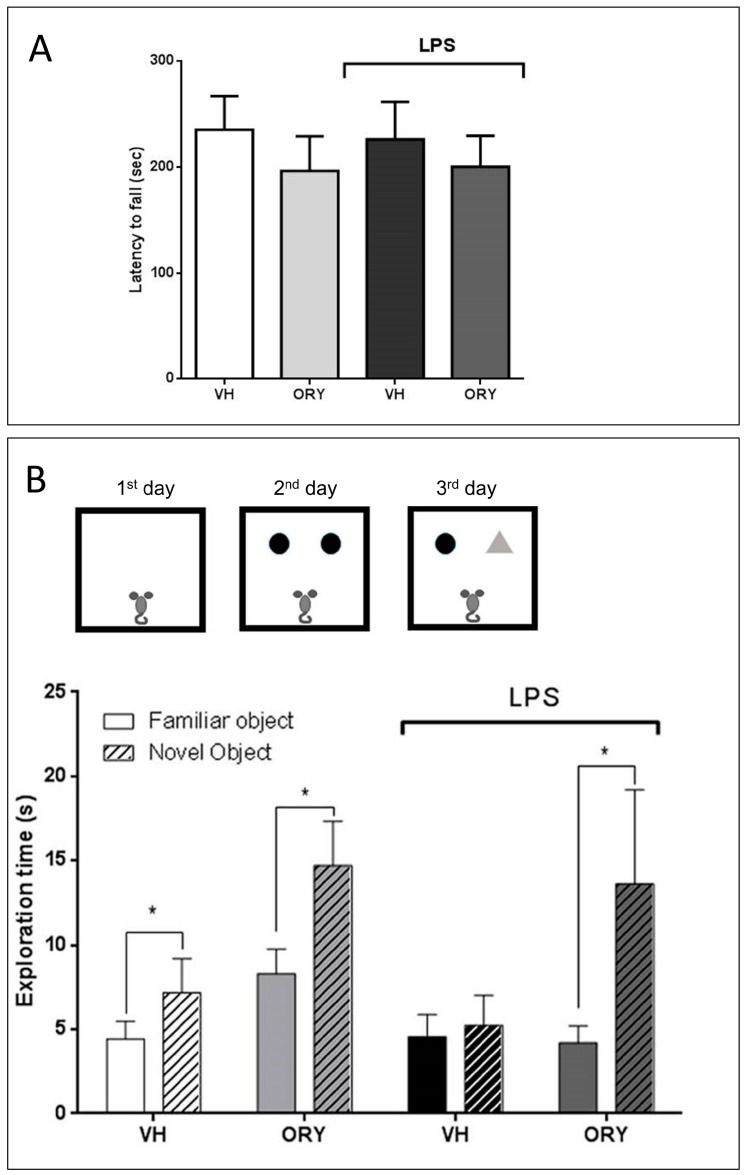
Motor performances of mice (**A**). Graphic scheme of the novel object recognition (NOR) test protocol and data of time spent exploring the familiar and novel objects are reported. For all experimental conditions, the full color bars represent the familiar object, while the patterned bars represent the novel object (**B**). Data are expressed as the mean ± S.E.M. Two-way ANOVA tests with Sidak’s multiple comparisons test were used to test statistical significance (* *p* < 0.05 vs. the familiar object). VH: vehicle; ORY: γ-oryzanol; LPS: lipopolysaccharide.

Since LPS induces acute and widespread inflammation accompanied by an outbreak of pro-inflammatory mediators, prior to the cognitive test, mobility performance was evaluated by using the rotarod test (Figure 2A). Motor activity was comparable between the two groups, showing no differences in term of latency to fall (F_treatment_ (3, 33) = 0.24, *p* = 0.87). In addition, LPS did not affect mobility in the ORY group or in the VH group.

The effect of ORY on long-term memory was then assessed by using the novel object recognition test (NOR) (Figure 2B). This test included three phases: habituation (day 1), the acquisition trial (day 2), and the actual test (day 3). In the acquisition phase, the time spent exploring the two identical objects was similar for ORY and VH mice treated or not treated with LPS (data not shown). When one of the two objects was substituted with a new one (third day of the trial), both ORY and VH mice showed higher interest for the novel object compared to the familiar object. On the contrary, mice treated with LPS lost their ability to distinguish the new object from the familiar one and indiscriminately explored both. Interestingly, mice pre-treated with ORY and subsequently injected with LPS were able to distinguish the novel object, spending more time exploring it than the familiar one (F_interaction_ (3, 66) = 1.15, *p* = 0.33; F_treatment_ (3, 66) = 2.92, *p* < 0.05; F_objects_ (1, 66) = 6.99, *p* < 0.05).

### 3.3. γ-oryzanol is Able to Counteract the Increase of LPS-dependent Proinflammatory Markers Increase in the Hippocampus

**Figure 3:**
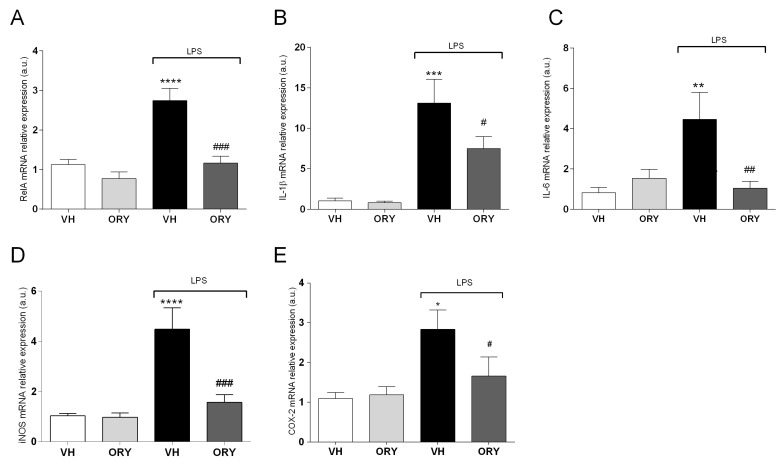
Graphic representation of quantitative RT-PCR data obtained from RNA extracted from the hippocampi of VH, ORY, LPS, and ORY+LPS mice. Data are expressed as the fold change of the target gene (RelA in (**A**), IL-1β in (**B**), IL-6 in (**C**), iNOS in (**D**) and COX-2 in (**E**)) normalized to the internal standard control gene (β-Actin). Data are shown as the mean ± S.E.M. One-way ANOVA tests with Newman–Keuls post-test were used to determine statistical significance, * *p* < 0.05, ** *p* < 0.005, *** *p* < 0.0005 and **** *p* < 0.0001 vs. VH, # *p* < 0.05, ## *p* < 0.005 and ### *p* < 0.0005 vs. LPS. VH: vehicle; ORY: γ-oryzanol; LPS: lipopolysaccharide.

After behavioural tests, mice were sacrificed, and hippocampi were collected for molecular analysis of the inflammatory mediators (Figure 3). As expected, LPS treatment increased the mRNA expression of both cytokines, IL-1β and IL-6, as well as of RelA (coding gene of Nuclear Factor-κB (NF-κB) p65 protein), the inducible nitric oxide synthase (iNOS), and cyclooxygenase-2 (COX-2), when compared to the control group (VH-treated mice). ORY-pre-treated mice challenged with LPS showed a statistically significant reduction of mRNA expression for all the mediators analyzed: RelA (F_treatment_ (3, 33) = 17.7, *p* < 0.0001), IL-1β (F_treatment_ (3, 33) = 11.5, *p* < 0.0001), IL-6 (F_treatment_ (3, 33) = 5.6, *p* < 0.005), iNOS (F_treatment_ (3, 33) = 12.92, *p* < 0.0001), and COX-2 (F_treatment_ (3, 33) = 4.36, *p* < 0.05). 

### 3.4. γ-oryzanol Upregulates the Expression of Second-generation Antioxidant Enzymes in the Hippocampus

**Figure 4:**
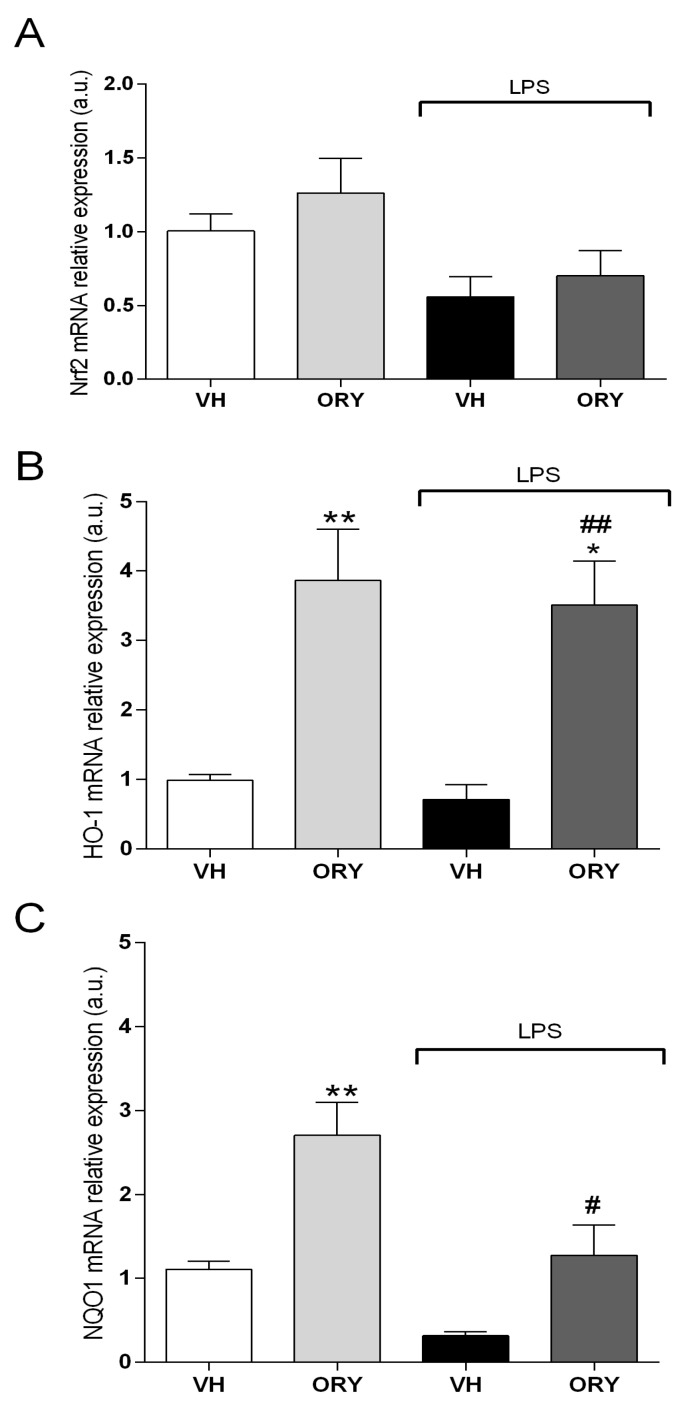
Graphic representation of quantitative RT-PCR data obtained from RNA extracted from VH, ORY, LPS, and ORY+LPS mice hippocampi. Data are expressed as the fold change of target genes (Nrf2 in (**A**), HO-1 in (**B**) and NQO1 in (**C**)) normalized to the internal standard control gene (β-Actin). Data are shown as the mean ± S.E.M. One-way ANOVA tests with the Newman–Keuls post-test were used to determine statistical significance, * *p* < 0.05 and ** *p* < 0.005. VH, # *p* < 0.05, and ## *p* < 0.005 vs. LPS. VH: vehicle; ORY: γ-oryzanol; LPS: lipopolysaccharide.

It is well known that the Nrf2-ARE phase II enzymes play a key role in the antioxidant and anti- inflammatory response [21]. In establishing the effects of ORY on this pathway, the mRNA expressions of Nrf2, HO-1, and NQO1 were evaluated (Figure 4). Nrf2 mRNA expression did not significantly change among the different experimental groups (F_treatment_ (3, 33) = 3.23, *p* = 0.305). On the other hand, chronic ORY treatment notably increased HO-1 (F_treatment_ (3, 33) = 9.34, *p* < 0.005) and NQO1 (F_treatment_ (3, 33) = 11.92, *p* < 0.0001) gene expression compared to VH. Interestingly, ORY was able to induce the gene expression of Phase II antioxidant enzymes, even after pro-inflammatory stimulation, although the magnitude of the increase was lower respect to ORY alone. Indeed, HO-1 and NQO1 mRNA expression was statistically higher in ORY-treated mice challenged with LPS compared with animals from the reference group (VH plus LPS) (HO-1: *p* < 0.005; NQO1: *p* < 0.05).

## 4. Discussion

In this study, we demonstrated that chronic treatment with ORY restored cognitive impairment and exerted anti-inflammatory effects in a mouse model of neuroinflammation. In particular, we found a correlation between ORY treatment and improvement of long-term memory with the reduction of the inflammatory response in mice exposed to LPS. In addition, ORY alone was able to increase the antioxidant response through the induction of the expression of Phase II antioxidant enzymes, such as HO-1 and NQO1, suggesting a “para-hormetic” action against future insults.

It is well recognized that neuroinflammation induced by LPS or by polyinosinic:polycytidylic acid (poly I:C) are known experimental paradigms to explore the mechanisms related to deficits in behavioural performance [22,23]. Moreover, the upregulation of pro-inflammatory cytokines induced by LPS has been associated with altered redox homeostasis and cognitive impairment [24,25,26,27]. In accordance with other authors [28,29,30], 48 h after a single dose of LPS (10 µg/mouse), we observed evident inflammation at the hippocampal level without an effect on locomotor activity. In addition, as far as cognitive aspects are concerned, LPS-treated mice lost the ability to distinguish between the novel object and the familiar object in the NOR test. Interestingly, mice pre-treated with ORY and then challenged with LPS showed some improvement in cognitive performance, as they spent more time interacting with the novel object than with the familiar one. This result is in line with that found by Mamiya and colleagues, who demonstrated that pre-treatment with ferulic acid, the main γ-oryzanol metabolite, rescued a deficit in memory and cognitive functions in the NOR test [31].

How ORY prevented LPS-induced cognitive impairment is a main point of interest. We hypothesized that such effect could be associated with its well-known antioxidant and anti-inflammatory effects. Among the mediators of inflammation, nuclear factor-κB (NF-κB) plays a central role [32,33]. It consists of a family of transcription factors that includes RelA (p65), RelB, c-rel, p50, and p52 [32], which mediate many physio-pathological pathways, including immune responses, inflammation, neurodevelopment, and proliferation [33,34,35,36,37]. The reduction of RelA (p65) by ORY in LPS treated mice could be an upstream event in the anti-inflammatory response of this rice compound. In fact, other studies have demonstrated that cycloartenyl ferulate, a phytosteryl ferulate of ORY, and ORY itself reduce the transactivation of pro-inflammatory genes throughout the inhibition of NF-κB p65 nuclear translocation, both in macrophages and inflamed peripheral tissues [38,39]. It is known that pro-inflammatory genes, including IL-1β, IL-6, iNOS, and COX2, recognized to be under the control of NF-κB p65 [40,41], are also involved in several diseases involving strong cognitive and memory alterations [27,42,43,44,45]. In particular, IL-1β modulates memory processes related to fear and stress [46], and when upregulated, it is associated with memory alterations [27]. Sparkman and colleagues showed that the expression of IL-6, induced by LPS treatment, alters long-term memory performance, increasing the expression of IL-1β and TNF-α [47,48]. In addition, elevated iNOS and COX-2 levels were associated with poor cognitive test performance and neurodegenerative disorders [44,49]. Our data confirmed a strong upregulation of all these mediators at the hippocampal level after LPS treatment. On the other hand, ORY pre-treatment prevented the increase in the LPS-induced mRNA levels of the cytokines IL-1β and IL-6 and the enzymes iNOS and COX2 in the mouse hippocampus.

The Nrf2 system is strictly involved in the modulation of inflammatory pathways [21]. Nrf2 activation regulates the expression of cytoprotective and antioxidant genes [50]. Under basal conditions, Nrf2 forms a complex with Kelch-like ECH-related protein 1 (Keap1), resulting in the degradation and inactivation of Nrf2 via ubiquitination. After a harmful stimulus, including LPS, Nrf2 separates from Keap1 and translocates into the nucleus where it can bind to the regulatory antioxidant response element (ARE) to induce the expression of enzymes such as glutathione-S-transferase, HO-1, and NQO1 [51]. Recently, in an in vitro model, we demonstrated that ORY is able to trigger the Nrf2 pathway in terms of the upregulation of Nrf2 mRNA and protein expression, translocation into the nucleus, and induction of the Nrf2-dependent defence genes [14]. Our new data show that in vitro treatment with ORY was not sufficient to increase Nrf2 mRNA expression. However, indirect evidence of the effects of ORY on Nrf2 pathway activation was given by its effects on HO-1 and NQO1 gene expression. Indeed, we observed hippocampal mRNA upregulation of HO-1 and NQO1 in mice that were chronically treated with ORY, which was still maintained upon LPS-induced neuroinflammation. These two enzymes, which regulate redox homeostasis and inflammatory responses [52,53], are even involved in cognitive performance. Indeed, HO-1 signalling was found to increase memory and learning abilities in mice [54] and to attenuate cognitive deficits induced by amyloid beta in an animal model of Alzheimer-like pathology [55]. A recent study also reported that ferulic acid improves cognitive abilities through the activation of the HO-1 pathway in the rat hippocampus [56]. NQO1, which is typically co-expressed with HO-1, is a cytosolic homodimeric flavoprotein that catalyses the two-electron reduction of quinones, preventing the formation of reactive semiquinones. Chhetri and colleagues recently reported an association between Alzheimer’s disease-related cognitive decline and the downregulation of the Nrf2 system, which also involves NQO1 [57]. The fact that chronic treatment with ORY increased the steady state levels of HO-1 and NQO1, exerting protective action against noxious stimuli, could also explain its cognitive improvement in the NOR test (ORY vs. VH mice).

Lastly, the reciprocal crosstalk existing between NF-κB p65 and Nrf2/HO-1 pathways [58] could explain the effects of ORY on the LPS mice model. It is noteworthy that the LPS-induced increase in p65 subunit expression was significantly higher in nuclear extracts of Nrf2 knockout mice than in wild-type mice [58]. Moreover, Nrf2-dependent HO-1 increased expression inhibits NF-κB p65 activity [59]. The inhibition of NF-κB activity was also induced by Keap1, the activator of Nrf2, through ubiquitination [60]. Therefore, on the basis of our results, it seems that ORY acts on these transcriptional factors, favoring antioxidant mediators and providing protection from inflammatory stimuli, and this condition promotes better cognitive performance. The central role of Nrf2 pathways in cognitive processes can be corroborated by some recent data on their capacity to regulate adult hippocampal neurogenesis by participating in the maintenance of synaptic networks [61,62,63]. Further studies are needed in this area.

## 5. Conclusions

*Oryza sativa* L., the millennial plant that feeds the entire globe has emerged as not only an important nutrient supply, but can also be considered as a food for healthy brains. In fact, rice bran is an abundant source of γ-oryzanol which, as demonstrated, induces molecular changes in the brain that are able to prevent cognitive impairment related to neuroinflammation, preserving memory and cognitive “homeostasis”.

## Figures and Tables

**Table 1:** Primers used for q-PCR.

Genes	Primer Sequences
Rel-A (p65)	f-5′-TTCCTGGCGAGAGAAGCAC-3′; r-5′-AAGCTATGGATACTGCGGTCT-3′
Inducible nitric oxide synthase (iNOS)	f-5′-CAGCTGGGCTGTACAAAC-3′; r-5′-CATTGGAAGTGAAGCGTTTCG-3′
Cyclooxygenase 2 (COX-2)	f-5′-GCAAATCCTTGCTGTTCCAACCCA-3′; r-5′-TTGGGGATCCGGGATGAACTCTCT-3′
Interleukin 1 beta (IL-1β)	f-5′-GCTTCAGGCAGGCAGTATC-3′; r-5′-TAATGGGAACGTCACACACC-3′
Interleukin 6 (IL-6)	f-5′-CCTACCCCAATTTCCAATGCT-3′; r-5′-TATTTTCTGACCACAGTGAGGAAT-3′
Heme oxygenase 1 (HO-1)	f-5′-TGAAGGAGGCCACCAAGGAGG-3′; r-5′-AGAGGTCACCCAGGTAGCGGG-3′
NAD(P)H dehydrogenase (quinone) 1 (NQO1)	f-5′-AGGATGGGAGGTACTCGAATC-3′; r-5′-TGCTAGAGATGACTCGGAAGG-3′
Nuclear factor (erythroid-derived 2)-like 2 (NRF2)	f-5′-AGCCCCATTCACAAAAGACA-3′; r-5′-GAAGTCATCAACAGGGAGGTTA-3′
Actin (β-act)	f-5′-AGCCATGTACGTAGCCATCC-3′; r-5′-CTCTCAGCTGTGGTGGTGAA-3′

**Table 2:** Body weight and food intake. VH: vehicle; ORY: γ-oryzanol.

	Body Weight (g)		Food Intake (g)	
Days of Treatment	VH	ORY	VH	ORY
0	41.9 ± 1.9	44.7 ± 1.3	/	/
7	40.4 ± 1.8	41.7 ± 1.2	19.3 ± 0.7	17.4 ± 0.5
14	40.0 ± 1.8	41.7 ± 1.1	19.8 ± 0.5	20.0 ± 0.4
21	41.3 ± 1.9	42.5 ± 1.2	22.9 ± 1.0	22.6 ± 0.5
